# Polyunsaturated Fatty Acids Influence LPS-Induced Inflammation of Fish Macrophages Through Differential Modulation of Pathogen Recognition and p38 MAPK/NF-κB Signaling

**DOI:** 10.3389/fimmu.2020.559332

**Published:** 2020-10-06

**Authors:** Qingfei Li, Kun Cui, Mengjiao Wu, Dan Xu, Kangsen Mai, Qinghui Ai

**Affiliations:** ^1^ Key Laboratory of Aquaculture Nutrition and Feed (Ministry of Agriculture) & Key Laboratory of Mariculture (Ministry of Education), College of Fisheries, Ocean University of China, Qingdao, China; ^2^ Laboratory for Marine Fisheries Science and Food Production Processes, Qingdao National Laboratory for Marine Science and Technology, Qingdao, China

**Keywords:** large yellow croaker, LPS, polyunsaturated fatty acids (PUFAs), inflammatory responses, signaling transduction, immunomodulatory effects

## Abstract

Polyunsaturated fatty acids (PUFAs) not only serve as essential nutrients but also function as modulators of the immune response in marine fish. However, their immunomodulatory mechanism is poorly understood given that the underlying regulation of the innate immune response in fish has not been fully elucidated. Hence, study of the innate immunity of fish could help elucidate the mechanism by which PUFAs affect the fish immune response. Here, we used combined transcriptome analysis and *in vitro* experimentation to study the mechanism of LPS-induced inflammation. Transcriptome profiling indicated that LPS elicited strong pro-inflammatory responses featuring high expression levels of pathogen recognition receptors (PRRs) and cytokines along with the activation of NF-κB and MAPK signaling pathways. The transcription factor p65 alone could increase the transcription of *IL1β* by binding to the promoter of *IL1β*, and this promoting effect disappeared after mutation or deletion of its binding sites. We then examined the effects of PUFAs on the levels of gene expression and the abundance of proteins of critical kinases associated with LPS-induced inflammation. We found that LA exerts pro-inflammatory response while ALA, EPA, and DHA induced anti-inflammatory effects by modulating the expression of PRRs, phosphorylation of IKK and p38, and the nuclear translocation of p65. Overall, this study advances our understanding of the regulatory mechanisms by which PUFAs regulate LPS-induced inflammation in a non-model fish species.

## Introduction

Currently, the aquaculture industry is facing global shortfalls in the supply of fish oil. With its relatively considerable output, lower price, and relatively high content of polyunsaturated fatty acids (PUFAs), vegetable oil is a promising alternative to fish oil (rich in ω-3 PUFAs). However, replacing fish oil with vegetable oil in fish diets can result in substantial variation in the dietary composition of fatty acids, altering the immunological responses of fish to infections (1). Because of the potency and economic benefits of PUFAs, study of the immunomodulatory effects of PUFAs has become a major focus of research, and a central goal of this research is to improve the health of fish and their resistance to pathogens. ω-6 PUFAs are widely known to exert pro-inflammatory effects, while ω-3 PUFAs are known to have anti-inflammatory properties through a wide range of mechanisms, including (i) differential modulation of the expression of pathogen recognition receptors (PRRs) (2); (ii) regulation of signaling pathway activities; and (iii) production of lipid mediators that modify the functions of immune cells. Despite an abundance of seemingly robust data, a majority of studies have only demonstrated “associations” but not “cause and effect,” and in some cases, results are even “confliction” (3). As a result, the mechanism by which individual fatty acids affect fish inflammation remains unclear.

Generally, elucidating the detailed mechanism governing fish immune responses is important, as the establishing mechanisms lay the molecular foundation for understanding the immunomodulatory roles of PUFAs (4). Recently, advances in genomic research in fish have benefited studies of the molecular mechanisms of fish immunity, as new genomic techniques have been widely applied in fish immune studies (5, 6). Nevertheless, few experimental validations have been made to support the raised working model. An *in vitro* approach is considered a promising tool for exploring the molecular events related to immunity for advantages including its simplification, convenience, and specificity together with exclusion of cell-cell interactions and environmental interference (7). Macrophages are the major immune effector cells in fish and are capable of detecting and responding robustly to various pathogen-associated molecular patterns (PAMPs) to execute protective functions against intruding pathogens (8). Functional immune responses can be recapitulated *in vitro* using macrophages derived from fish, and these results have been validated through observation of a “highly directed response and more generalized state of activation” (9–11). Hence, the combination of transcriptome sequencing and *in vitro* assays should provide sensitive and efficient analyses for exploring how pathogen recognition, signaling transduction, and gene expression are orchestrated during the response of fish to specific stimuli.

Large yellow croaker is an economically important marine fish in China, but its susceptibility and vulnerability to bacterial disease is considered a main constricting factor for its expansion in the aquaculture industry. In aquatic environments, infectious bacteria with the capacity to reproduce independently of their host can cause severe inflammation, increased morbidity, and even mortality (5, 12). Moreover, the ubiquitous replacement of fish oil in the diets of large yellow croaker negatively alters their immune capacity against disease and their resilience to stress (13). In addition, the availability of the draft genome of large yellow croaker permits study of gene function and the molecular mechanisms underlying biological responses. Thus, large yellow croaker could serve as an excellent model for studying the immunity-PUFA interactions in fish. The objective of this present study was to elucidate the mechanism by which the innate immune system of fish responds to bacterial infection. In doing so, this study sheds light on the mechanism underlying PUFAs-mediated inflammation in the macrophages of large yellow croaker. These insights advance our understanding of host-pathogen relationships in fish and contribute to the development of nutritional strategies mediated by PUFAs for treatment against bacterial infections.

## Materials and Methods

### Fish, Macrophage Culture, and Treatments

Large yellow croaker (weight 750 ± 50 g) of the Daiqu strain were purchased from a commercial fish farm in Ningbo, China, and were kept in a flow-through water system at 20–22°C. Macrophages were isolated from fish head kidney and maintained according to a modified procedure described previously (14). Macrophage-rich cells were adjusted to 2.0×10^5^ cells/mL and seeded in cell culture plates (Nunc, Denmark). After overnight incubation at 26°C, unattached cells were removed after two vigorous washes, and the resultant macrophage monolayer was primed in DMEM/F12 medium supplemented with 5% FBS (Gibco, USA) for the experiments described below.

**Experiment 1:** In transcriptome sequencing, macrophages were stimulated by LPS (from *Escherichia coli* O55:B5, Sigma, USA) for 2 h, and cells cultured in DMEM/F12 medium were used as controls. Cells were prepared in triplicate for sequencing. **Experiment 2:** In kinetics of gene expression and protein synthesis associated with LPS-induced innate immune response, macrophages were exposed to LPS stimulation for 1, 3, 6, and 18 h before harvest for RNA or protein extraction. **Experiment 3:** In inhibition assay, macrophages were exposed to 30 min of incubation with 2.5 μM NF-κB inhibitor BAY11-7082 (InvivoGen, USA), 5 μM MEK inhibitor PD98059 (Sigma, USA), p38 MAPK inhibitors SB3580 (Sigma, USA), or JNK inhibitor SP600125 (MCE, USA) prior to 6 h of stimulation with LPS. Cells were then later sampled for RT-PCR analyses. **Experiment 4:** In immunomodulatory effects of PUFAs on the LPS-induced inflammatory response in macrophage, macrophages were incubated with 100 μM PUFAs, namely linoleic acid (LA), linolenic acid (ALA), eicosapentaenoic acid (EPA), and docosahexaenoic acid (DHA), for 12 h before LPS stimulation for 3 h (for western blot analysis) and 6 h (for gene expression). Notably, the final working concentrations of LPS, PUFAs, and inhibitors were determined when significant changes in the expression of representative genes were observed and when cell viability was not compromised through time and dose-dependent assays.

### RNA Extraction, cDNA Library Construction, and Sequencing

TRIzol reagent (Takara, Japan) was used for RNA extractions as per the manufacturer’s instructions. RNA integrity and purity were assessed by 1% agarose gel electrophoresis and NanoDrop UV spectrophotometry analysis, respectively. According to the NEBNext Ploy(A) mRNA Magnetic Isolation Module (NEB) protocol provided by Novogene, RNA samples were used to construct RNA-seq libraries followed by quantification with a Qubit 2.0 Fluorometer and Agilent 2100 bio-analyzer. Then libraries were sequenced on an Illumina NovaSeq6000 platform and 150bp pair-end reads were generated.

### Differential Gene Expression Analysis

After removing reads containing adaptor sequences, reads with an N, and low-quality reads (> 50% of bases with a quantity score Q-phred ≤ 20), clean reads were subsequently aligned to the genome and mapped to the coding sequences of the large yellow croaker genome using Hisat2 (v2.0.5). The DESeq2 R and edgeR R package were used to perform DEGs analysis, respectively. Gene expression levels were calculated based on the FPKM method (expected number of Fragments Per Kilobase of transcript sequence per Millions base pairs sequenced). Differences between the LPS treatment and the control were assessed by a strict Poisson distribution algorithm. DEGs were defined when the adjusted P-value ≤0.05 determined by DESeq2 and edgeR, and absolute value of log_2_ (fold change) ≥ 1.5 were obtained.

### Gene Ontology (GO) and Kyoto Encyclopedia of Genes and Genomes (KEGG) Analysis

All DEGs were mapped to the GO database (http://www.geneontology.org) (15). For GO enrichment analysis, all P-values were Bonferroni corrected. A corrected P-value of 0.05 was used as the threshold for determining the significant enrichment of gene sets. The large-scale molecular datasets generated by genome sequencing were processed by the KEGG database (http://www.genome.jp/kegg). We used the clusterProfiler R package to test for the statistical enrichment of DEGs in KEGG pathways.

### Quantitative RT-PCR

RT-PCR was performed using a Roche LightCycler^®^ 96 system (USA) and SYBR Premix Ex Taq (Tli RNaseH Plus) (Takara, Japan) as described previously (16). The sequence-specific primer sets were designed based on the coding sequences of identified genes in the large yellow croaker genome (**Supplementary Table 1**). In the current study geNorm algorithm was used to determine the most stable reference genes (17). We found that the most stable genes in the samples determined by geNorm analysis with an acceptable M value < 0.5 were β-actin = 18S rRNA < ubiquitin < GAPDH.Validation of the geNorm reference gene rankings for each sample type was confirmed following the re-analysis of data using BestKeeper software (18). Hence, β-actin was the most stable transcript across all of the samples and was used as the internal control. Each sample was run in triplicate, and the fold change was calculated using the comparative 2-ΔΔCT method (19).

### Western Blot Analysis

Cells were harvested by adding RIPA reagent (Solarbio, China) along with supplementation of protease inhibitors (Thermo Fisher Scientific, USA). Nuclear protein was extracted using NE-PER Nuclear and Cytoplasmic Extraction Reagents (Thermo) per the instruction manual. Protein concentration was evaluated by BCA protein assays (Beyotime Biotechnology, China) for adjustments. Western blot experiments were performed as described previously (20). Denatured protein was separated by 10% sodium dodecyl sulfate polyacrylamide gel electrophoresis (SDS-PAGE) and was then transferred to polyvinylidene fluoride membranes (Millipore, USA). After blocking with 5% skimmed milk at room temperature for 2 h, the membrane was incubated with primary antibodies overnight at 4°C. Finally, the membrane was incubated with secondary antibodies for 2 h, which were then visualized by an Electrochemiluminescence kit (Beyotime Biotechnology). Signals generated on the western blot were recorded using a densitometer to scan the film. Image J software (USA) was then used to quantify the generated bands. The rabbit polyclonal antibodies targeting IKKβ (Cell Signaling Technology, Cat# 2678, RRID : AB_2122301) and lamin B1 (Santa Cruz Biotechnology, Cat# sc-374015, RRID : AB_10947408) as well as rabbit p-IKKα/β mAb (Cell Signaling Technology, Cat# 2697, RRID : AB_2079382) were diluted at 1:1000. Rabbit monoclonal antibodies against p65 (Cell Signaling Technology, Cat# 8242, RRID : AB_10859369), p38 (Cell Signaling Technology, Cat# 8690, RRID : AB_10999090), p-p38 (Cell Signaling Technology, Cat# 9215, RRID : AB_331762), JNK (Cell Signaling Technology, Cat# 9252, RRID : AB_2250373), and p-JNK (Cell Signaling Technology, Cat# 4668, RRID : AB_823588) were diluted at 1:2000. Rabbit ERK mAb (Cell Signaling Technology, Cat# 4695, RRID : AB_390779) and p-ERK mAb (Cell Signaling Technology, Cat# 4370, RRID : AB_2315112) were diluted at 1:4000. Mouse anti-GAPDH mAb (ZSGB-Bio, Cat# TA-08, RRID : AB_2747414), secondary antibodies including HRP-labeled goat anti-rabbit IgG polyclonal antibody (ZSGB-Bio, Cat# ZB-2301, RRID : AB_2747412) and HRP-labeled goat anti-mouse IgG polyclonal antibody (Beyotime Biotechnology, #A0216, RRID : AB_2860575) were diluted at 1:5000.

### Plasmid Construction and Luciferase Reporter Assays

Specific primers were designed based on large yellow croaker genomic data to construct luciferase reporters and expression plasmids (**Supplementary Table 2**). The primers for truncated promoters were designed to amplify the shortened sequences extending from ATG translation start codon to different sites upstream of IL1β. Primers for the mutant Luc-IL1β promoters were designed using a site-directed mutagenesis method (Takara, Japan). All generated PCR products were inserted into pGL3-basic plasmid (Promega, USA) as reporter plasmids using the homologous recombination method provided by ClonExpress II One Step Cloning Kit (Vazyme Biotech, China). To construct expression plasmids of transcription factors (TFs), including P50, P65, C-JUN, C-FOS, and STAT4, PCR products were ligated into pcDNA3.1 plasmids (Invitrogen, USA) using the method described above. All plasmids for transfection were prepared using the EasyPure HiPure Plasmid MiniPrep Kit (TransGen Biotech, China) and were verified by sequencing at Sangon Biotech Co., Ltd. (Shanghai, China).

For luciferase assays, reporter plasmids, expression plasmids, and pRT-TK renilla luciferase plasmids were co-transfected into HEK293T cells. After 24 h of transfection, cells were harvested and detected by the Dual-Luciferase Reporter Assay System (TransGen Biotech, China). The firefly and Renilla luciferase activities were read by a SpectraMax i3x multifunctional detection station (Molecular Devices, USA). Transfections were performed in triplicate.

### Chromatin Immunoprecipitation Assay

To determine the *in vitro* DNA-binding activity of p65 with IL1β promoter, HEK293T cells were transfected with plasmids containing IL1β promoter and plasmids containing FLAG (as vector control) or p65-Flag followed by 36 h of culture at 37°C. Next, cells were harvested after cross-linking of the target protein and genomic DNA in 1% formaldehyde. Immunoprecipitation of chromatin (ChIP) assay was performed according to the protocol provided by a commercial ChIP kit (Thermo). TheDYKDDDDK Tag (D6W5B) rabbit mAb (Cell Signaling Technology, Cat# 14793, RRID : AB_2572291) was used to immunoprecipitate and isolate the target from other nuclear components, and rabbit mAb IgG (Cell Signaling Technology, Cat# 3900, RRID : AB_1550038) served as the control in this study. The resultant purified chromatin was amplified by PCR for 40 cycles with specific primer pairs for the detection of genomic regions corresponding to the promoter and the control.

### Statistical Analysis

All raw data were subjected to one-way ANOVAs followed by Tukey’s multiple-range tests, and the results were presented as means ± SEM. The threshold for statistically significant difference between means was P < 0.05.

## Results

### Transcriptome Analysis of LPS-Treated Macrophages

Illumina pair-end sequencing technology generated two transcriptomes for the control group and the LPS-treated group, resulting in a total of 75,241,451 and 69,006,809 clean reads, respectively. Data were then mapped to the L. crocea genome with mapping coverage falling within 85%–86%. Raw sequencing reads data have been deposited in NCBI under the accession number SRX7427103–SRX7427108. Two well-established statistical analysis methods (DESeq2 and edgeR) were applied to identify DEGs. Compared with the control group, a total of 465 DEGs consisting of 415 up-regulated genes and 50 down-regulated genes were identified by DEseq2 method, while 554 up-regulated genes and 84 down-regulated genes totaling 638 DEGs were found by edgeR method in the LPS-treated group (**Supplementary Figure 1**).

GO analysis showed that DEGs were highly enriched into two major functional categories: biological process and molecular function. The biological category was characterized by immune responses and immune system process terms. The molecular function category was abundant in cytokine receptor binding, receptor binding, tumor necrosis factor receptor binding, chemokine activity, and chemokine receptor binding (**Supplementary Figure 1**). KEGG analysis revealed that LPS-stimulated DEGs were associated with the following pathways: C-type lectin receptor signaling pathway, Toll-like receptor signaling pathway, Cytokine-cytokine receptor interaction, RIG-I-like receptor signaling pathway, etc. (**Supplementary Figure 1**).

### Annotation of DEGs Associated With the Innate Immune Response

Functional annotation further classified DEGs into three clusters: pattern recognition receptor; adaptors and signal transduction protein; and chemokines, cytokines, and receptors **(** **Supplementary Table 3**).

### Expression Kinetics of Innate Immunity-Related Genes in Response to LPS

**Figure 1:**
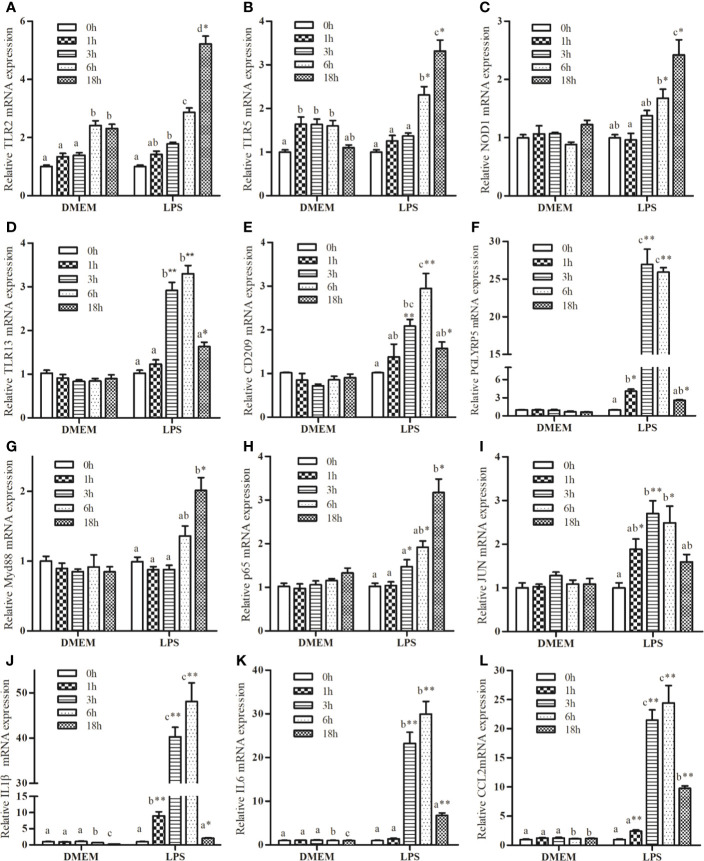
Kinetics expression of *TRL2* **(A)**, *TRL5* **(B)**, *NOD1* **(C)**, *TLR13* **(D)**, *CD209* **(E)**, *PGLYRP5* **(F)**, *MyD88* **(G)**, *P65* **(H)**, *JUN* **(I)**, *IL1β* **(J)**, *IL6* **(K)**, and *CCL2* **(L)** in macrophages of large yellow croaker following 50 μg/mL LPS stimulation. Values are presented as mean ± SEM (n=4). Means with different superscript letters are significantly different (*P* < 0.05, Tukey’s test) relative to the 0 h time point within the same treatment. *P <0.05 and **P <0.01 indicate significant difference compared with the control at each time point.

Several genes were selected for further analysis based on their functional annotations. Following exposure to LPS, the mRNA expressions of *TLR2*, *TLR5*, and *NOD1* increased significantly up to 18 h relative to the control group (**Figures 1A–C**). Increase in the expression of *TLR13*, CD209, and *peptidoglycan recognition protein 5* (*PGLYRP5*) were sustained up to 6 h, followed by a marked drop at 18 h (**Figures 1D–F**). LPS promoted a significant increase in *myeloid differentiation factor 88* (*MyD88*) expression (**Figure 1G**), but not in *TIR-domain-containing adapter-inducing interferon-β* (*TRIF*) transcription (data not shown). LPS markedly increased the expression of *P65* and *JUN* (**Figures 1H, I** **)**. In addition, LPS significantly up-regulated the expression of *IL1β*, *IL6*, and *CCL2* compared with the control group (**Figures 1J–L**).

### Activation of NF-κB and MAPK Signaling Pathways Induced by LPS

**Figure 2:**
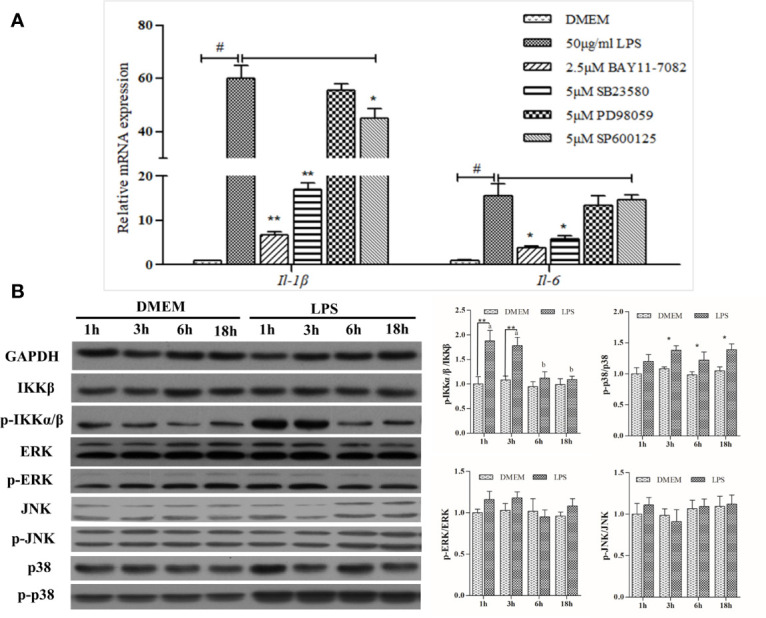
Analysis of NF-κB and MAPK signaling activation. **(A)** Effects of various inhibitors on mRNA expression of LPS-induced inflammation related genes in large yellow croaker head kidney macrophages. Data are expressed as mean ± SEM (n=4). ^#^ *P <* 0.05 indicates significant differences relative to the DMEM group as the negative control; *P < 0.05 and **P < 0.01 indicate significant differences compared with the LPS treatment as the positive control. **(B)** Western blot analysis of relevant protein expression in the macrophages. Data are expressed as A.U. of the western blots, and signaling activation is depicted as the ratio of phosphorylated protein to total protein. Data are presented as mean ± SEM (n = 3). *P < 0.05, **P < 0.01 when compared with the control group at each time point. Means with different superscript letters are significantly different (*P* < 0.05, Tukey’s test) within the same treatment.

BAY11-7082, the NF-κB pathway inhibitor, significantly down-regulated IL1β and IL6 transcription by~ 90% and ~80%, respectively, relative to the LPS positive treatment. ERK inhibitor PD98059 showed no inhibitory effects, whereas SB23580, the p38 inhibitor, was able to reduce LPS-induced expression of *IL1β* and *IL6* substantially. The expression-promoting effects of LPS were partially attenuated by JNK inhibitor SP600125, which had a less pronounced effect than BAY11-7082 and SB23580 in decreasing *IL1β* expression (**Figure 2A**).

LPS could significantly upregulate the activity of the NF-κB pathway, as levels of phosphorylated-IKK increased 1 h and 3 h post-stimulation and then declined gradually thereafter. Despite this slight increase, no significant differences were noted in either ERK or JNK pathway after LPS stimulation, whereas levels of phosphorylated-p38 protein were significantly increased (**Figure 2B**). Overall, LPS strongly activated NF-κB and MAPK signaling pathways featuring phosphorylation cascades.

### p65 Alone Controls Transcription of IL1β by Binding to Its Promoter

**Figure 3:**
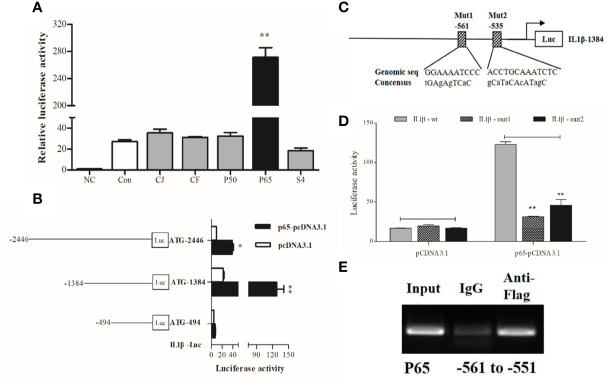
Transcriptional analysis of large yellow croaker IL1β. **(A)** HEK293T cells were co-transfected with *IL1β* promoter construct plasmids, pRL-TK renilla luciferase plasmid, and with expression plasmids of transcription factors, including C-JUN (CJ), C-FOS (CF), P50, P65, STAT4 (S4). The negative control (NC) consisted of empty plasmids (pGL3-basic), and cells transfected with *IL1β* luciferase reporter plasmids were the control (Con). **(B)** A series of truncated promoter plasmids containing various lengths of the promoter regions starting from ATG and **(C)** mutant promoter reporters with specific site-directed mutagenesis in the *IL1β* promoter (ATG-1384) were constructed. **(D)** HEK293 cells were co-transfected with truncated or mutant promoter plasmids and p65-pcDNA3.1 expression plasmids, cells transfected with luciferase reporter and empty vector pcDNA3.1 represented the control. At 36 h post-transfection, luciferase activity was measured. **(E)** ChIP assay identified p65 binding sites in the promoter of *IL1β*. The luciferase activities are normalized to the NC group and presented as means ± SEM (n = 3). *P < 0.05; **P < 0.01 when compared with the control.

*IL1β* was chosen as the representative effector for the pro-inflammatory response because of its high expression upon LPS stimulation. Dual-luciferase reporter assays showed that p65 alone could significantly activate *IL1β* luciferase activities, which was highlighted by the significant increase (~10 fold) in *IL1β* promoter activity compared with the control group (**Figure 3A**).

To locate the putative binding sites of the critical transcriptional factor p65 in the promoter of *IL1β*, we constructed luciferase plasmids containing truncated sequences and mutated putative binding sites in the promoters according to the computational prediction made by the Jasper (21) and Promo (22) algorithms. Truncated promoter analyses revealed that the binding sites of p65 were located from 494 to 1384 bp upstream from the start codon ATG of *IL1β* (**Figure 3B**). Furthermore, a significant decline in promoter activity was observed after the predicted binding sites were mutated, indicating that the distant p65 binding site (p65 site 1, -561) in the *IL1β* luciferase reporter ATG-1384 was essential for transcriptional activity (**Figures 3C, D** **)**. Next, ChIP assays coupled with PCR data showed that fragments of DNA sequences amplified by specific primers contained the binding site of p65 in the *IL1β* promoter (**Figure 3E**).

### Fatty Acids Differentially Regulated LPS-Induced Inflammation through MAPK and NF-κB Pathways in Macrophages

**Figure 4:**
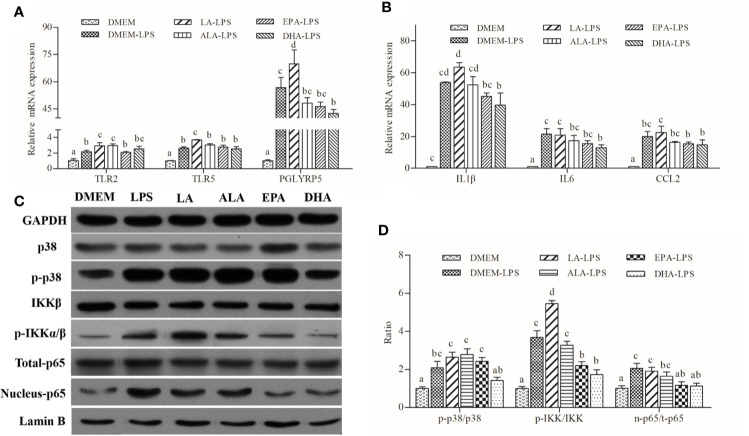
Effects of PUFAs on the innate immune response of large yellow croaker macrophages. **(A, B)** Expression of *TLR2*, *TRL5*, *PGLYRP5*, *IL1β*, *IL6*, and *CCL2* in macrophages of large yellow croaker by LPS (50 μg/mL) stimulation after incubation with PUFAs for 12 h. **(C, D)** The ratios of p-IKKα/β to IKKβ, p-p38 to p38, nucleus p65 to total p65 were determined; GAPDH or Lamin B were used as the total and nucleus reference protein, respectively. Data are expressed as A.U. of the western blots and are depicted as the ratio of target protein to reference protein. All data are presented as mean ± SEM (n= 3). Means with different superscript letters are significantly different (*P* < 0.05, Tukey’s test).

LA had pro-inflammatory effects by increasing LPS-induced expression of pro-inflammatory cytokines, including *IL1β* and *CCL2* (**Figure 4A**). ALA, EPA, and DHA appeared to possess anti-inflammatory functions; DHA exerted the strongest inhibitory effects followed by EPA and ALA. Patterns of expression of cytokines correlated well with those of PRRs genes, namely *TLR2*, *TLR5*, and *PGLYRP5* (**Figure 4B**). Furthermore, western blot analysis indicated that LA treatment increased the abundance of phosphorylated p38 and IKKα/β with concomitant increases in nuclear p65 content. Conversely, ALA, EPA, and DHA had pronounced inhibitory effects, and the inhibitory effect of DHA on the LPS-induced phosphorylation of IKKα/β and the unclear translocation of p65 was stronger than that of EPA and ALA. Specifically, DHA appeared to exert its inhibitory effect on the synthesis of phosphorylated p38 induced by LPS, while ALA and EPA showed no effects of phosphorylated p38 synthesis (**Figures 4C, D** **)**.

## Discussion

Bacterial diseases are one of the most important problems in current aquaculture; fish tend to rely more on their innate responses to combat invading bacteria because of their evolutionarily less-well-developed adaptive immune function (23). Innate immunity is the first line of defense against microbial pathogens, and the study of innate immunity to bacteria has focused heavily on the mechanisms by which immune cells deal with LPS, the conserved PAMP of Gram-negative bacteria (24). In mammals, LPS can be well recognized by toll-like receptor 4 (TLR4) (25). Immune signals are then transmitted downstream through MyD88 dependent and TRIF-dependent pathways, which activates a series of key TFs including NF-κB, interferon regulatory factors (IRFs), and mitogen-activated protein kinases (MAPKs), leading to the induction of pro-inflammatory cytokines, chemokines, and antimicrobial responses (20, 26). Fish share similar components of the immune system as mammals and thus possess innate immune responses similar to mammals. However, given that fish occupy a different portion of the evolutionary tree from mammals, accumulating evidence suggests that some detailed mechanisms are specific to fish (23). Unlike mammals, most fish lack TLR4 receptors according to the genomic data; even though TLR4 has been found in some fish, such as zebrafish, the function of LPS recognition is missing because of the absence of the costimulatory molecules MD2 and CD14-like constituents in mammals (27, 28). Overall, fish immunology is less well studied compared with mammals; consequently, the general principles underlying signal generation and the mediation of downstream signaling cascades in the immune regulatory network have yet to be fully elucidated.

Cytokines are biologically potent immune molecules secreted by stimulated immune cells that act to protect against pathogens (29). Irrespective of the type of immune cells, cytokine profiles released from activated cells primarily depend on the type of stimulus (30). In the current study, large yellow croaker head kidney macrophages could respond strongly to LPS stimulation, resulting in an increase in the mRNA transcripts of *IL1β*, *IL6*, and *CCL2*. Similar patterns of gene expression have also been described in rainbow trout (*Oncorhynchus mykiss*) (10) and Atlantic cod (*Gadus morhua* L.) (31). The impacts of PUFAs on the inflammation of macrophages have been extensively investigated both *in vitro* and *in vivo*, and these studies have generally shown that LA is the typical n-6 PUFA eliciting pro-inflammatory effects while ALA, EPA, and DHA possess anti-inflammatory properties (13, 32, 33). These findings are consistent with the results of the present study. We found that the reduction in the expression of pro-inflammatory genes in cells pretreated with DHA was more pronounced than that observed in other groups following LPS exposure. This finding is consistent with data obtained from human macrophages (34) and fish hepatocytes by our team (unpublished data).

The initiation of the innate immune response depends on the ability of the host to sense various PAMPs in pathogens through PRRs. Previous *in vivo* studies have suggested that the transcriptional levels of TLRs are increased after flagellin, poly(I:C), peptidoglycan (PGN), and pure LPS challenge in large yellow croaker, and flagellin and peptidoglycan had the strongest ability to stimulate *TLR2* (35). This observation is roughly similar to the present result showing that LPS induced higher levels of *TLR2* and P*GLYRP5* mRNA expression in large yellow croaker head kidney macrophages. One possibility is that the crude LPS used in the present experiment was contaminated with the TLR2 and PGRPs ligands flagellin and PGN from bacteria (5, 10). In addition, a recent study has shown that NOD1 was able to identify LPS (36); thus, the NLRs in teleost were considered partially responsible for LPS recognition. Here, LPS appeared to stimulate the transcription of NLRs, suggesting the role of fish NLRs in interactions with LPS. Given that fatty acids can affect the immune response by altering TLRs in large yellow croaker (20), we examined whether PUFAs could affect inflammation by PRR-mediated pathways. Pretreatment of LPS-induced macrophages with LA induced the expression of *TLR2*, *TLR5*, and *PGLYRP5*, whereas ALA, EPA, and DHA decreased these mRNA transcripts. Accumulating evidence in mammals has revealed that PUFAs can modulate PRR-mediated inflammation through a variety of strategies (37). However, existing literature on teleost PRRs has primarily focused on transcriptional responses to dietary factors, but few studies examined the function of signaling transduction. Further research is needed to fill this gap.

Typically, the transcription of target genes stems from the specific transduction of signaling cascades (38). Teleost NF-κB and MAPK pathways have been shown to govern the transcription of a variety of cytokine genes induced by LPS (25, 39, 40). Our results were consistent with these observations, as a cohort of signal transducers consisting of NF-κB pathways components, mitogen-activated protein kinase kinase kinase 8 (MAP3K8) were activated upon LPS stimulation. We next identified the roles of both pathways in pro-inflammatory responses and found that the inhibition of NF-κB and p38MAPK dampened the expression of *IL1β* and *IL6*. Furthermore, increased levels of phosphorylated IKKα/β and p38 MAPK proteins induced by LPS were also indicative of activation of the NF-κB and MAPK pathways, respectively. LPS did not increase the level of phosphorylated ERK at any of the assayed time, which was comparable to the results obtained on macrophages of salmon (41) and trout (42). Although no significant difference in phosphorylated JNK was detected, the expression of *IL1β* and *IL6* mRNA was suppressed after treatment with the JNK inhibitor SP600125. These observations altogether reinforce the previous conclusion that the p38 module plays an important role in inflammatory responses, that JNK can regulate the transcription of inflammatory genes directly or indirectly, and that ERK activity favors, but is not strictly necessary for, LPS-induced cytokine transcription in fish (42).

Activation of signaling pathways results in the nuclear translocation of central TFs that control gene expression through their direct or indirect interactions with binding sites in the promoters of target genes (43). As the downstream target of the NF-κB pathway, p65 is an important subunit of NF-κB, and the ap1 complex consisting of the TFs C-JUN and C-FOS is the downstream target of MAPK signaling. Here, we focused the regulatory predictions of TFs on the expression of *IL1β*, which was selected to be the representative target gene for its responsiveness (i.e., high levels of expression) to LPS stimulation. In mammals, both p65 and p50 appear to function as positive regulators in the transcriptional expression of *IL1β* (44). However, our study in fish suggested that p65 alone could trigger the transcription of *IL1β*, which was confirmed by a ChIP assay that confirmed the binding of p65 to DNA sequences of *IL1β* promoter. AP1 was reported unlikely to be effective because of the absence of its binding sites within *IL1β* promoter regions; nevertheless, AP1 might contribute by interacting with other TFs (45). This is supported by the present observation that the AP1 complex failed to affect the promoter activity of *IL1β*. Thus, the transcription of *IL1β* in fish might be a consequence of the synergistic actions of NF-κB and p38MAPK signaling combined with the nuclear translocation of p65.

Fatty acids appear to partially affect the inflammatory response *via* their capacities to modulate the activities of pivotal signaling pathways and the nuclear translocation of certain TFs (46). Pretreatment of ALA, EPA, and DHA had a promoting effect on the LPS-induced phosphorylation of IKKα/β and the impaired nuclear translocation of p65, whereas pretreatment of cells with LA had an opposite effect. Notably, DHA was the only PUFA that could also suppress the phosphorylation of p38, which might reflect the fact that it possessed the strongest anti-inflammatory properties among the PUFAs tested in this study. This finding is consistent with our previous study on the hepatocytes of large yellow croaker (unpublished data). However, the more specific mechanism underlying the immunomodulatory effects of fatty acids remains unclear. Numerous mammal studies have shown that PUFAs can affect the expression of inflammatory genes in a variety of manners, including post-transcriptional (47) and post-translational modifications (48), protein-to-protein interactions (49), macrophage polarization (20), and the production of lipid mediators (50). The immunology of fish has been traditionally less well understood compared with the immunology of mammals because there have been fewer studies conducted in fish. Therefore, these areas will likely be the focus of future research.

**Figure 5:**
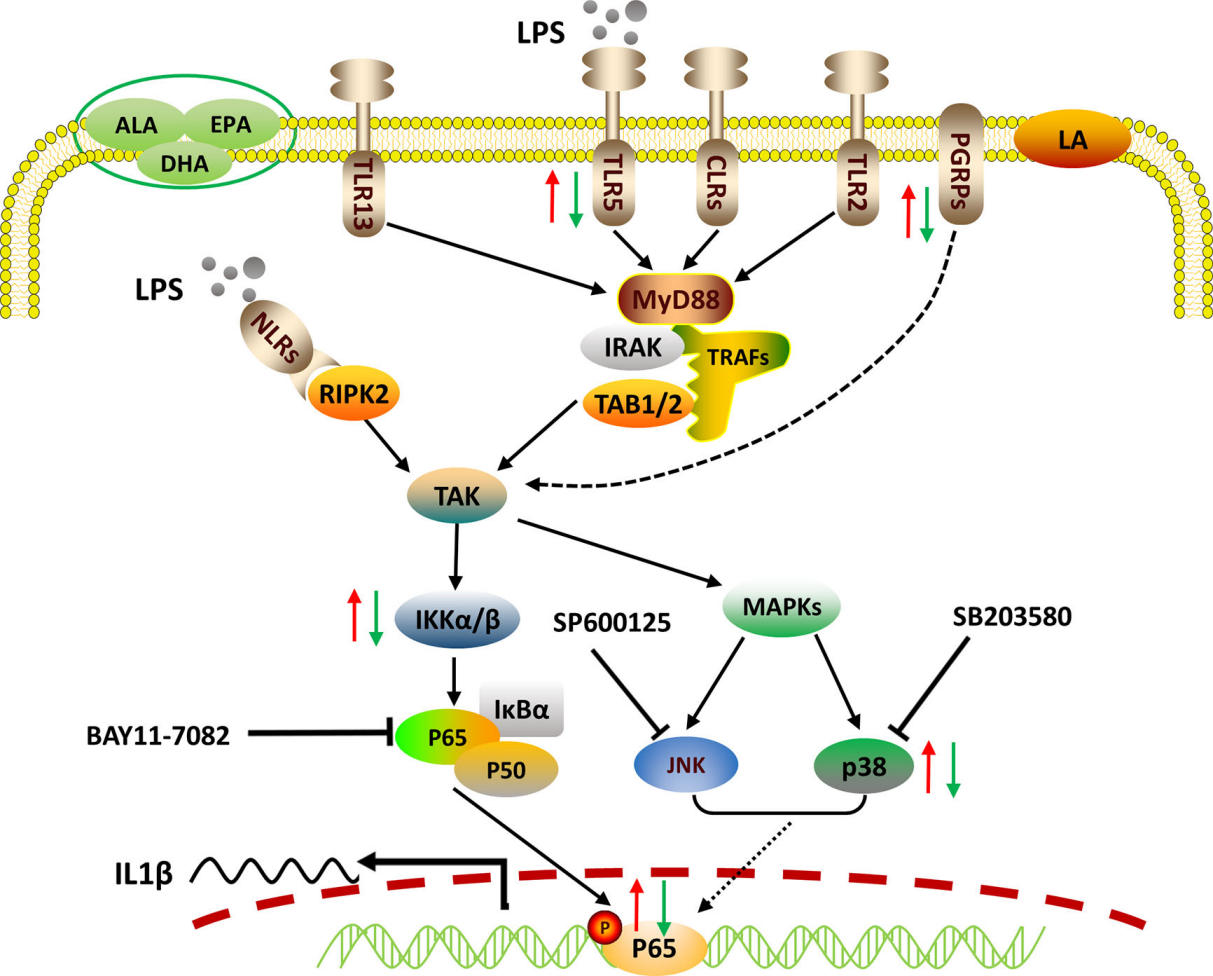
Proposed working model of how PUFAs regulate mRNA expression of the representative pro-inflammatory gene *IL1β*. Crude LPS is recognized by specific PRRs, including TLRs, NLRs, and PGRPs of fish macrophages. Triggered immune signals are transmitted downstream to activate NF-κB and MAPK pathways, leading to the nuclear translocation of p65, which controls the transcription of *IL1β* by binding to its promoter. PUFAs can differentially regulate the LPS-induced pro-inflammatory responses *via* pathogen recognition, signaling activation, and pro-inflammatory gene expression. The red arrows indicate the enhancing effects by LA and the green arrows show the suppressing effects exerted by ALA, EPA, and DHA.

In sum, we reported the LPS-stimulated transcriptome of large yellow croaker using an *in vitro* macrophage model. LPS induced the transcription of pro-inflammatory cytokines through pathogen recognition and activation of MAPK and NF-κB signaling pathways. We also confirmed that p65 alone could up-regulate the transcription of *IL1β* by targeting the specific response elements within its promoter. Grounded in those findings, PUFAs were found to differentially regulate the gene expression of PRRs, the activities of MAPK and NF-κB signaling, as well as the nuclear translocation of p65, thereby exerting differential immunomodulatory effects (**Figure 5**). Our findings advance our understanding of fish innate immunity against bacterial infections and contribute to the development of nutritional strategies for improving the health of cultured fish.

## Data Availability Statement

The datasets presented in this study can be found in online repositories. The names of the repository/repositories and accession number(s) can be found in the article/**Supplementary Material**.

## Ethics Statement

The animal study was reviewed and approved by Institutional Animal Care and Use Committee of Ocean University of China.

## Author Contributions

QA and QL designed the experiment. QL and KC conducted the research. QL, MW, and DX analyzed the data. QL wrote the manuscript. KM and QA revised the article. All authors contributed to the article and approved the submitted version.

## Funding

This research was supported by the National Science Fund for Distinguished Young Scholars of China (Grant NO. 31525024), Key Program of National Natural Science Foundation of China (Grant NO. 31830103), Ten-thousand Talents Program (Grant NO. 2018-29), and the Agriculture Research System of China (Grant NO. CARS-47-11).

## Conflict of Interest

The authors declare that the research was conducted in the absence of any commercial or financial relationships that could be construed as a potential conflict of interest.
