# Supplementary material for: Polyunsaturated Fatty Acids Influence LPS-Induced Inflammation of Fish Macrophages Through Differential Modulation of Pathogen Recognition and p38 MAPK/NF-κB Signaling
Source: Front Immunol. 2020 Oct 6;11:559332. doi: 10.3389/fimmu.2020.559332 (PMC7572853; doi:10.3389/fimmu.2020.559332)
Supplement: Supplementary file 1 [file DataSheet_1.pdf]

Supplementary Material

Supplementary Figure 1. Transcriptional analysis of LPS-treated macrophages of large yellow croaker using the DESeq2 and edgeR statistical model, respectively. (**i**) Volcano plot of differential expressed genes (when |log2 (fold change) |≥ 1.5 were obtained) of two treatments based on DESeq2 (A1) and edgeR (A1) methods. The X-axis indicates the value of $\log_2$ fold change and the Y-axis represents the significance of differential expression. The up-regulated genes and down-regulated genes are labeled by red dots and green dots, respectively. (**ii**) Diagram description of Gene Ontology enrichment of DEGs based on DESeq2 (B1) and edgeR (B2) methods. All the DEGs fall into two major categories: biological process and molecular function. The X-axis represents various gene functions, the Y-axis indicates $-\log_{10}$ (padj) values with significant differences. (**iii**) Scatter diagram of enriched pathways for DEGs based on DESeq2 (C1) and edgeR (C2) methods. In this scatter diagram, the top 20 pathways are listed, and rich factor is the ratio of DEGs in this pathway to all the genes in this pathway. The X-axis corresponds to rich factor of pathway, and the Y-axis indicates different pathways. The size of the dots displays gene number ranging from 2 to 10, and padj values are represented by the color classification.

Supplementary Table 1. Primer pair sequences for quantitative real-time PCR.

Supplementary Table 2. Primers for gene cloning and plasmid construction.

Supplementary Table 3. Innate immune-related differently expressed genes found in LPS-treated transcriptome based on the DEseq2 and EdgeR statistical models, respectively.

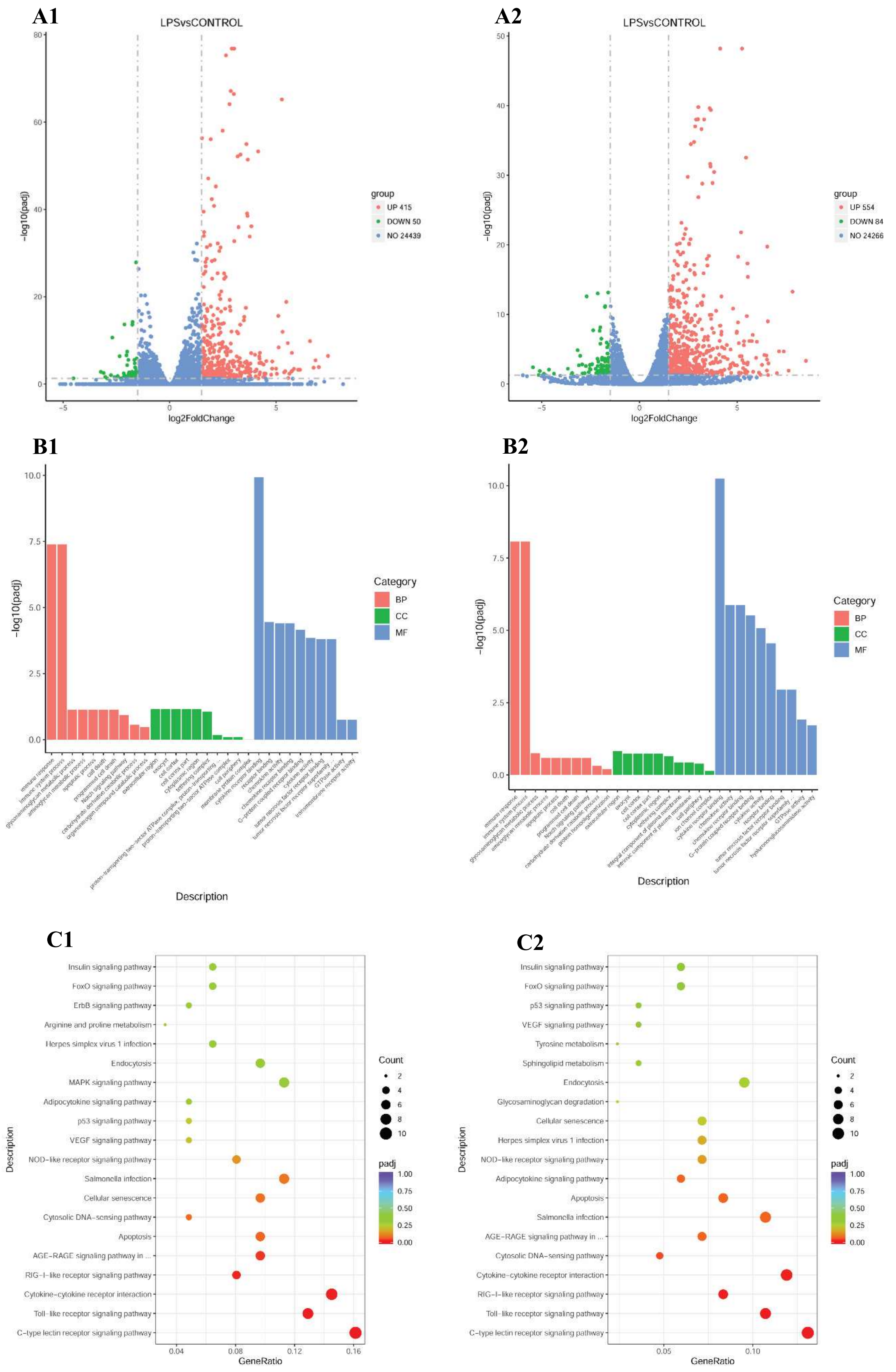

A1
LPSvsCONTROL
-log10(padj)
log2FoldChange
group
UP 415
DOWN 50
NO 24439
A2
LPSvsCONTROL
-log10(padj)
log2FoldChange
group
UP 554
DOWN 84
NO 24266
B1
-log10(padj)
Description
Category
BP
CC
MF
B2
-log10(padj)
Description
Category
BP
CC
MF
C1
Insulin signaling pathway
FoxO signaling pathway
ErbB signaling pathway
Arginine and proline metabolism
Herpes simplex virus 1 infection
Endocytosis
MAPK signaling pathway
Adipocytokine signaling pathway
p53 signaling pathway
VEGF signaling pathway
NOD-like receptor signaling pathway
Salmonella infection
Cellular senescence
Cytosolic DNA-sensing pathway
Apoptosis
AGE-RAGE signaling pathway in ...
RIG-I-like receptor signaling pathway
Cytokine-cytokine receptor interaction
Toll-like receptor signaling pathway
C-type lectin receptor signaling pathway
Description
GeneRatio
Count
padj
C2
Insulin signaling pathway
FoxO signaling pathway
p53 signaling pathway
VEGF signaling pathway
Tyrosine metabolism
Sphingolipid metabolism
Endocytosis
Glycosaminoglycan degradation
Cellular senescence
Herpes simplex virus 1 infection
NOD-like receptor signaling pathway
Adipocytokine signaling pathway
Apoptosis
Salmonella infection
AGE-RAGE signaling pathway in ...
Cytosolic DNA-sensing pathway
Cytokine-cytokine receptor interaction
RIG-I-like receptor signaling pathway
Toll-like receptor signaling pathway
C-type lectin receptor signaling pathway
Description
GeneRatio
Count
padj

Supplementary Table 1. Primer pair sequences for quantitative real-time PCR

Primer	Forward (5'-3')	Reverse (5'-3')	Fragment (bp)	PCR efficiency (%)	Accession NO.
TRL2	GTCATAAACGGCAGGGTGTT	AGCGTCATTTCAGTCAGCAG	104	98	XM_027282648.1
TRL5	CACCCTGGCAAGACATCCAA	CGGTGTGACCGTGGACTATG	183	101	XM_019267725.2
TLR13	GGACTCACCACCATCCAATC	ATCCAATGAAACACCCGTCT	109	102	NM_001303396.1
CD209	TGAGATGGTGGAGAGGGTAGT	GTCTGCAGTTTGGATGTGAATTTCT	210	97	XM_027282515.1
MyD88	GAGGATGGTGGTGGTGATTT	GGTGAGGAAGCGTAAGATGC	166	99	XM_010753272.3
PGLYRP5	GGAGCACATACCAAAGGCCA	TGTGGTACCCCTCAGTTGTG	237	97	XM_010733573.3
NOD1	ATCGCTGTGATGAATGTGGT	CATGATGAAGGAGGTGTCCC	93	98	XM_019279273.2
IL-1β	CAATCTGGCAAGGATCAGC	GGACGGACACAAGGGTACTAA	95	102	XM_010736551.3
IL-6	CGACACACCCACTATTTACAAC	TCCCATTTTCTGAACTGCCTCT	102	99	XM_010734753.3
CCL2	GCAGTCGCCCGATTCCTT	CCACTTTGCATCAGGATTGGC	167	99	XM_019262171.2
P65	ACTAACCTCTCCGAGCCCAT	TCATCTGGTTCAGCAGGCAG	107	98	XM_027288016.1
JUN	GTCGATCGTGGGACTTGTGT	CGTCGTAAAACGTCGCTTCC	100	98	XM_010741494.3
GAPDH	GGCTGCCAAGGAGTAAACCA	GGGGTCGACTATCTGAAGGC	145	97	XM_010743420.3
18S rDNA	GGGGTCCGAAGCGTTTACTT	CGGCCGTCCCTCTTAATCAT	150	98	NC_040020.1
ubiquitin	AGGCTGTGCATTCCTGACTT	CGACGTCGGACCACCTTAAA	148	101	XM_019278697.2
β-actin	GACCTCACAGACTACCTCATG	TGTTGTAGGTGGTCTCGTGGA	292	101	XM_027284923.1

Abbreviations: TLR, Toll-like receptor; MyD88, Myeloid differentiation primary response 88; PGLYRP5, peptidoglycan recognition protein 5; NOD1, nucleotide binding oligomerization domain containing 1; IL1β, interleukin 1 beta; IL6, interleukin 6; CCL2, C-C motif chemokine 2; P65, putative transcription factor p65 homolog; JUN, C-Jun-amino-terminal kinase-interacting protein 1.

Supplementary Table 2. Primers for promoter cloning and plasmid construction

Primer	Sequence (5'-3')
IL1β-pGL3-F1	*GGCTCGAGATCTGCGATCTAAGTA*TCCATTGTAGAAACCTGCTGTGA
IL1β-pGL3-F2	*GGCTCGAGATCTGCGATCTAAGTA*GGAGCCGTTCTTCACTCAC
IL1β-pGL3-F3	*GGCTCGAGATCTGCGATCTAAG*TACCATGTGATGTGTATATAACCTG
IL1β-pGL3-R	*CTTTACCAACAGTACCGGAATGCCA*AAAGAGATCCGCAGGGAAGAC
IL1β-Mut1-F	GCTGAACtGAgAgTCaCATGTGATGTGTATATAACCTGCAAATCT
IL1β-Mut1-R	TGtGAcTcTCaGTTCAGCATGAAGTGGGATGATG
IL1β-Mut2-F	gCaTaCAcATagCACAGAGGGGCAGAACAACACTG
IL1β-Mut2-R	CTGTGctATgTGtAtGcTATATACACATCACATGGGATTTTCCG
STAT4-pcDNA3.1-F	*AACTTAAGCTTGGTACCGAGCTCG*ATGAGCCAGTGGAAACAGATTCA
STAT4-pcDNA3.1-R	*GAATTCCACCACACTGGACTAGTG*TTAATCTGAATATGGAGAACTCAT
FOS-pcDNA3.1-F	*AACTTAAGCTTGGTACCGAGCTCG*ATGATGTTTCCCGTTTTCAACA
FOS-pcDNA3.1-R	*GAATTCCACCACACTGGACTAGTG*TTAAAGAGCCAGAAGGGTGGG
JUN-pcDNA3.1-F	*AACTTAAGCTTGGTACCGAGCTCG*ATGATCGCACGGATCACAAGAGAA
JUN-pcDNA3.1-R	*GAATTCCACCACACTGGACTAGTG*CTAGAAAGCTTGGAGCTGCTGCGT
P50-pcDNA3.1-F:	*AACTTAAGCTTGGTACCGAGCTCG*ATGTGTTTGTTTGTTCAAACGGACGCG
P50-pcDNA3.1-R:	*GAATTCCACCACACTGGACTAGTG*TCACGCCGTGGAGAGCTCCACC
P65-pcDNA3.1-F:	*AACTTAAGCTTGGTACCGAGCTCG*ATGGCGGATGTGTACGGATG
P65-pcDNA3.1-R:	*GAATTCCACCACACTGGACTAGTG*TTAAGTTGGGTGTCCTGACA
ChIP- IL1β-F	AGTACAGGATTTCATCATCCCACT
ChIP- IL1β-R	GTGCGTTTACCTGTTAAGGTCAGT

Abbreviations and GenBank Accession number: P50, nuclear factor kappa B subunit 1, XM_027280560.1; p65, putative transcription factor p65 homolog, XM_027288016.1; JUN, Jun proto-oncogene, XM_010741494.2; FOS, proto-oncogene c-Fos, XM_010735240.3;STAT4, signal transducer and activator of transcription 4, XM_027280453.1. Sequences of homologous arms of vectors were shown in italic and mutated bases were indicated as lowercase letters.

Supplementary Table 3. Innate immune-related genes found in LPS-treated transcriptome

Description	Gene name	log_2ratio (LPS/Control)	P-value	log_2ratio (LPS/Control)	P-value
Statistical model		DESeq2		EdgeR	
**Pattern recognition receptor**					
toll-like receptor 5	TLR5	6.59	1.45E-17	6.54	2.22 E-23
CD209 antigen-like protein E	CD209E	6.12	2.04E-05	5.74	6.08E-09
peptidoglycan-recognition protein 5	PGLYRP5	6.02	2.07E-05	5.62	6.07E-09
**Adaptor and signal transducers**					
NFKB inhibitor delta%2C transcript variant X1	NFKBID	5.88	2.31E-08	4.59	1.31E-22
TNFAIP3 interacting protein 1-like		5.27	2.50E-69	5.25	5.07E-53
BCL3 transcription coactivator	BCL3	3.03	1.68E-81	3.01	1.89E-44
tumor necrosis factor-like		2.90	3.59E-05	2.89	5.72E-10
v-rel avian reticuloendotheliosis viral oncogene homolog B	RELB	2.83	8.00E-21	2.81	2.03E-19
NF-kappa-B inhibitor alpha	IKBA	2.81	3.47E-68	2.80	7.30E-39
CYLD lysine 63 deubiquitinase	CYLD	2.58	1.72E-03	2.49	2.08E-04
nuclear factor kappa B subunit 2	NFKB2	2.53	9.75E-16	1.88	6.23E-56
TNFAIP3 interacting protein 1	TNIP1	2.50	6.19E-06	2.46	2.52E-07
transcription factor AP-1	AP1	2.31	4.39E-14	2.29	1.65E-13
nuclear factor of kappa light polypeptide gene enhancer in B-cells 2	NFKB2	2.30	8.69E-09	2.28	1.91E-10
mitogen-activated protein kinase kinase kinase 8	MAP3K8	2.22	1.74E-28	2.23	6.14E-32
BCL2 like 14	BCL2L14	2.10	9.23E-04	2.07	4.95E-08
somatomedin-B and thrombospondin type-1 domain-containing	SI:DKEY-	2.03	2.09E-04	1.99	9.77E-06
TNF superfamily member 13b	TNFSF13B	1.95	2.72E-03	1.92	4.88E-06
nuclear factor of kappa light polypeptide gene enhancer in B-cells	NFKBIAB	1.91	1.90E-13	1.90	2.51E-12
nuclear factor of kappa light polypeptide gene enhancer in B-cells	NFKBIE	1.75	4.39E-32	1.73	1.84E-18
suppressor of cytokine signaling 1a	SOCS1A	1.68	4.89E-29	1.66	3.97E-17
tumor necrosis factor b	TNFB	1.58	1.37E-15	1.56	1.52E-12
**Chemokine, Cytokine and receptor**					
chemokine (C-C motif) ligand 20a, duplicate 3	CCL20	5.38	1.01E-04	5.34	2.78E-08
interferon a3	IFNa3	4.77	4.52E-04	4.59	2.07E-04
interleukin-1 beta	IL1B	4.14	3.89E-18	4.17	3.52E-12

C-C motif chemokine ligand 17	CCL17	3.61	7.60E-59	3.59	3.76E-44
interleukin 17 receptor B	IL17RB	3.58	1.48E-20	3.55	5.34E-22
interleukin-12 subunit beta	IL12B	3.36	2.17E-07	3.37	5.83E-11
interleukin-8	IL8	3.29	2.11E-08	3.27	5.99E-16
tumor necrosis factor	TNF	3.22	1.29E-07	3.20	1.55E-14
interleukin-6	IL6	2.98	9.87E-08	2.96	2.04E-14
prostaglandin E synthase	PTGES	2.79	6.68E-08	2.80	8.85E-11
prostaglandin-endoperoxide synthase 2b	PTGS2B	2.44	4.48E-12	2.42	3.88E-12
C-X-C motif chemokine ligand 13	CXCL13	1.73	3.80E-17	1.71	3.43E-14
C-X-C chemokine receptor type 1		1.62	2.81E-13	1.60	1.95E-11
prostaglandin E2 receptor EP4 subtype	PTGER4A	-1.61	2.10E-10	-1.60	6.67E-13
interleukin-1 receptor-like 2		-1.75	1.79E-16	-1.77	3.05E-14
phosphoinositide-3-kinase, regulatory subunit 2 (beta)	PIK3R2	-3.02	1.88E-03	-2.93	3.88E-05
